# Establishment and Maintenance of Feline Pregnancy—A Comprehensive Review

**DOI:** 10.3390/ani15091249

**Published:** 2025-04-28

**Authors:** Sabine Schäfer-Somi

**Affiliations:** Clinical Center of Reproduction, University of Veterinary Medicine Vienna, Veterinärplatz 1, 1210 Vienna, Austria; sabine.schaefer@vetmeduni.ac.at

**Keywords:** cats, pregnancy, embryo, placenta

## Abstract

Cats are different from dogs, and many questions remain open concerning the establishment of pregnancy. In cats, as in dogs, no feto-maternal signaling leading to establishment of pregnancy is known. But as opposed to dogs, the placenta is a source of steroid hormones and corticotropin-releasing hormone (CRH). Scarce information is available on physiological mechanisms at the uterine level during early gestation; more studies are needed on lymphocyte subsets, feto-maternal crosstalk and other mechanisms leading to local immunosuppression, allograft acceptance and embryo nidation and invasion. Recent studies investigate the function of extracellular vesicles (EVs); however, there is no study on embryo- or endometrium-derived EV. During pregnancy, anti-Müllerian hormone (AMH) serum concentrations were found to be higher than in non-pregnant cats, and a recent study found that supraphysiological levels may lead to pregnancy loss; the function of AMH during pregnancy warrants investigation. Most information is available on corpus luteum development and function, showing some similarities to dogs. Some information on maintenance of feline pregnancy was obtained by ovariectomy (OE) or the use of endocrine disruptors, showing that OE does not lead to pregnancy loss in all cases, especially when performed after day 35; the variable effect is still not fully understood. Antiprogesterone, dopamine agonists and prostaglandins were used in different dosages and treatment schemes and showed variable effect during the second half of gestation, highlighting progesterone and prolactin as key hormones for the maintenance of gestation. Some events during early gestation are comparable with the canine species, even though they appear earlier, like the entrance of the zygote into the uterus and implantation; however, significant differences are present concerning the histomorphology of the placenta and, in a few cases, even the gross morphology as in some cats, where the zonary placenta does not completely surround the fetus. Sonographical monitoring of feline pregnancy requires knowledge of species-specific developmental steps and the differential appearance of fetal and maternal structures in comparison with dogs.

## Simple Summary

Many questions concerning feline pregnancy are still open; therefore, older and more recent scientific findings were collected and combined in a comprehensive review, highlighting what has been achieved so far and where more science is needed. More information is available on the cat cycle than on early pregnancy, the feto-maternal signaling, molecular changes inside the uterus and decidualization. Similarly, the mechanisms leading to parturition are not well investigated. Some events during early gestation are comparable with the canine species; however, significant differences are present concerning the endocrinology and histomorphology of the placenta and, in a few cases, even the gross morphology. Sonographical monitoring of feline pregnancy requires knowledge on the development and special appearance of fetal and maternal structures.

## 1. Introduction

Cats are different, and this concerns, among other things, the sexual cycle, endocrinology of pregnancy and parturition and the prerequisites for establishment of pregnancy. Good progress was achieved in the investigation of the feline estrus cycle and the neurohormonal regulation of ovulations. However, concerning establishment of pregnancy, many questions are open. Cats are seasonal, polyestrus breeders; the sexual cycle is regulated by light intensity and duration. During autumn, melatonin from the pineal gland reduces secretion of gonadotropins from the pituitary gland. Increasing daylight and intensity suppresses melatonin secretion, which induces an increase in gonadotropin secretion and consecutive follicular growth [1]. During estrus, serum estradiol concentration is maximal [1,2]; ovulations occur either spontaneously or are induced by repeated matings, and they occur approximately 24–48 h after ovulations [3,4]. Plasma progesterone concentration peaks by day 25–30 and then slowly decreases; in the second half of gestation, the main source of gestagens is the feline placenta until parturition at 65 days on average [2,5]. The early events following conception also differ from the canine species. Zygote formation, entrance into the uterus, nidation and implantation occur earlier than in dogs, even though the duration of pregnancy may be longer, lasting on average 66–67 days and up to 71 days in some cases [6,7]. In cats, as in dogs, the placenta is the only source of relaxin; however, contrary to dogs, the placenta in addition secretes progesterone, estrogens and even corticotropin-releasing hormone (CRH, [8,9]). Corpora lutea formation and function shows some similarities to the dog; however, while in dogs many questions concerning regulation of corpus luteus function could be answered [10], the differential regulation of CL regression in pregnant and non-pregnant cats still warrants investigation, even though some studies are available [11,12,13]. Furthermore, in cats, as in dogs, no feto-maternal signaling leading to establishment of pregnancy is known. Little is known about physiological mechanisms at the uterine level; histological changings were described until day 20 of gestation [14], but there is a lack of information on changes in lymphocyte subsets, feto-maternal crosstalk and other mechanisms leading to local immunosuppression, allograft acceptance and embryo nidation and invasion. Actual in vitro studies on ART investigate the production and secretion of extracellular vesicles by maternal and embryo tissue and their effects on embryos and endometrium during early gestation; the mechanisms show some similarity between species [15,16]. However, in cats, regulation of decidualization and the mechanisms leading to parturition are not sufficiently investigated. Finally, this review shall provide an overview on what is known about the establishment and maintenance of feline pregnancy.

## 2. Endocrinology of Early Pregnancy—What Is Special in Cats

In cats, mating leads to ovulations and the formation of corpora lutea (CLs), as well as a significant increase in serum progesterone concentrations during the first week after mating, starting around day 2–3 after mating. In parallel, a quick decline of estradiol during the first approximately 5 days takes place, with values remaining low during both pseudopregnancy and pregnancy; however, in pregnant cats, an increase thereafter and until day 15 was observed, with concentrations staying elevated until parturition; the placenta is an additional source of estradiol secretion [8,17]. The serum estradiol concentration varies between individuals, rendering this steroid hormone unsuitable for timing of parturition. During the first half of pregnancy, progesterone is produced by CLs; later, production and secretion of gestagens are performed by the placenta while the CLs regress [8]. Progesterone serum concentrations are higher in pregnant cats than in non-pregnant cats, especially during the first half of gestation [18]. Relaxin is produced by the syncytiotrophoblast; it is the only pregnancy-specific hormone, and the serum concentrations show a similar course as in dogs [19,20,21]. Prolactin is secreted by the anterior hypophysis, and concentrations increase towards parturition, staying elevated during lactation. Prostaglandin(PG)F2α secretion from the placenta also increases towards term, which is mirrored by an increase in the concentration of its metabolite PGFM during the three weeks before parturition, rising functionally during the last week; in dogs, this occurs later, 24–48 h before parturition [22].

## 3. Ovarian Activity During Pregnancy

### 3.1. Follicle Formation and AMH Secretion

In pregnant cats, follicle formation despite existing corpora lutea is a physiological observation [23]. In the first half of pregnancy, this frequently occurs, and these follicles may undergo ovulations. Follicle numbers were counted during different pregnancy stages and did not differ between stages [18]. In the pregnant cat, as in other mammals, the glycoprotein anti-Müllerian hormone (AMH), a member of the transforming growth factor-ß superfamily [24], is produced in ovarian preantral and early antral follicles. In cyclic animals, AMH inhibits excess recruitment of primordial follicles and decreases the sensitivity of FSH receptors on growing follicles [25,26,27]. In pregnant animals, the function is not well investigated. In one study, serum AMH concentration increased significantly until day 32–40, stayed elevated until day 46, and decreased towards term (*p* < 0.05). Serum AMH concentration was independent from number of fetuses [18]. Protein expression of AMH was highest in follicles and corpora lutea of pregnant cats between days 41 and 46 in comparison with other pregnancy stages and in comparison with interestrus and estrus cats (*p* ˂ 0.05). Receptors for AMH were found to be in close proximity to AMH, like in granulosa cells of small antral follicles and corpus luteum cells, and were always more positive signals than in non-pregnant cats; expression was remarkably strong in interstitial cells surrounding the follicles that underwent atresia [18]. In rats, this was suggested to be a result of suppression of follicular maturation [28]. Contrary to non-pregnant cats, no positive correlations between ovarian AMH, antral follicles and total antral follicles were assessed; however, between days 32 and 40, a significant, positive correlation between number of corpora lutea and ovarian AMH expression was determined. These observations are restricted to cats of a younger age of approximately 1 year, which is important to mention as AMH expression is age-dependent [29]. Finally, at present, the higher serum AMH concentration in pregnant cats in comparison with non-pregnant cats is supposed to be related to follicular activity during feline mid-pregnancy. Whether the corpus luteum actively secretes AMH, contributing to the increased serum concentration between days 32 and 46, warrants further investigation. Recently, injection of a feline AMH analog using an adeno-associated viral vector led to a >1000-fold increase in AMH concentration, which was detectable for 9 months. In pregnant cats, this induced pregnancy loss by fetal resorption at approximately 6–7 weeks of pregnancy [30]. Elevated AMH levels could, in conjunction with a disturbance of the hypothalamic–pituitary function, lead to alteration of placental steroid metabolism [31].

### 3.2. Corpus Luteum Formation and Function

In cats, matings may induce ovulations by neurohormonal stimulation [23,32] and are followed by corpora lutea (CLs) formation; however, in pregnant and non-pregnant cats, the time of CLs regression differs. Infertile matings are followed by a shortened luteal phase [23,32] and lower progesterone (P4) presence than in pregnant cats from day 10 to 12 on. Progesterone decreases from day 21 on [33] and becomes basal between days 25 and 40 after mating [2,33]. In pregnant cats, CL show signs of regression from day 38 to 39 and on, and the serum P4 values gradually decline until the end of pregnancy, becoming basal immediately after parturition [2,11,17]. Peak values of P4 are reached between days 11 and 30, with a gradual decline until parturition [17,34].

Valuable information is available concerning the intraluteal production and effects of estrogens, androgens and progesterone (for a review: [13]). In both feline pregnant and pseudopregnant CLs, the mRNA of steroidogenic enzymes cholesterol side-chain cleavage enzyme (cytochrome P450 family 11 subfamily A member 1[CYP11A1]), steroidogenic acute regulatory protein (stAR), 3β-hydroxysteroid dehydrogenase/Δ5-Δ4 isomerase (3ßHSD) as well as steroid 17-α-monooxygenase (CYP17A1) and subtypes 1 and 7 of 17β-hydroxysteroid dehydrogenase (HSD17B) were detected [35]. Furthermore, local mRNA and protein expression of estrogen, progesterone and androgen receptors was proven [12] Hormone concentration in the CL was independent from pregnancy and higher during formation of CLs than during regression [11,35]. Progesterone concentration in the corpus luteum was maximal during the maintenance stage of the CL and paralleled the serum P4 concentration [2,11,35]. Prostaglandins (PGs) and receptors were found to be expressed in the CLs of pregnant and non-pregnant cats. Expression of PG synthases PTGS2/COX2 and PTGES and the PG receptor PTGER4 was independent of the luteal stage [36]. In pregnant cats, the PGE:PGF2α ratio was maximal in the pre-implantation stage, while it stayed unchanged in non-pregnant cats. In pregnant cats, luteal regression is triggered by extra-luteal PGF2α of placental origin. Apoptotic factors contribute to the formation and regression of CL, irrespective of pregnancy [11].

## 4. Placental Hormone Secretion

In the feline placenta, biosynthesis and secretion of estradiol and progesterone was found. Steroidogenic enzymes 3ßHSD and stAR are localized in the decidual cells [8]. Placental P4 concentrations increase towards late gestation. Intraplacental steroidogenic enzymes show the same expression pattern as the hormones [21].

Biosynthesis and secretion of relaxin in the feline placenta is similar to dogs. Relaxin is secreted by the fetoplacental unit from day 20 of gestation on [19]. The placenta is the main source of relaxin; in other organs, mRNA expression was weak. The relaxin receptor (RXFP1) is mainly expressed in the pregnant uterus and placenta [20].

Placental prostaglandin (PG)FS protein was elevated at 2.5–3 weeks of gestation, and protein expression was detected in trophoblast cells and also at term in decidual cells; PGFS mRNA declined until parturition. PTGS2 mRNA was upregulated towards term; protein expression was detected in the trophoblast cells around blood vessels and decidual cells. Placental PGF2α content increased towards term, paralleled by plasma PGFM concentrations [22].

Only recently, expression of corticotropin-releasing hormone (CRH) mRNA and protein was found in the feline placenta, in larger decidual cells and fetal trophoblast cells, between days 21 and 35 of gestation. The hormone was additionally detected in maternal blood, increasing from early pregnancy to mid-pregnancy and reaching values resembling human concentrations during the early third trimester [9]. Expression of the specific receptor within the placenta and local functions of CRH warrants further investigation; the question remains as to whether CRH is necessary in cats for the establishment and maintenance of pregnancy, as in humans. The role of cortisol during feline pregnancy despite fetal organ maturation is also not sufficiently investigated.

## 5. Prerequisites for Feline Pregnancy Establishment

The mechanisms leading to immunotolerance of the fetal allograft are grossly unknown; embryonic and uterine secretion products and enzymes were supposed [37,38]. An earlier study found that a balance in the cell populations of Treg and TH17 cells is important [39]. Most probably, embryo and uterine secretion products contribute to the change in the uterine milieu, as in dogs. However, the details and timing may be different [40].

### Effect of Ovariectomy and Hormone Disruptors

Progesterone and prolactin are thought to be essential for successful pregnancy establishment, as administration of antiprogesterone or a dopamine agonist may lead to the termination of pregnancy at any gestational stage. But the impact of the ovaries is variable; after the formation of the placenta, they proved not to be required in some cases. In one study [41], ovariectomy (OE) was performed on Day 35, and abortion occurred in all cats. When OE was performed on day 40, abortion occurred in four-fifths of cats; OE on day 45 induced abortion in two-fifths and OE on day 50 in three-fifths of cats. In the remaining cats, normal delivery took place on days 63–69 of pregnancy. In another study, OE on Day 45 induced abortion in all cats [34].

Similarly, s.c. application of the synthetic PGF2α analog cloprostenol on three consecutive days from day 21 to 22 and on, failed to induce abortion in all cats, but when applicated from day 35 to 38 and on, one-seventh of cats aborted [42]. During a later study [43], the same protocol was used, resulting in no abortions when cloprostenol was administered from day 21 to 22 and on and in 1/10 abortions when injected from day 35 to 38 and on. Interestingly, when natural PGF2α (2 mg) was applied on day 33, all cats aborted despite normal placenta function and P4 values [34]. The same was found by [44], when natural PGF2α was injected after day 40. All injections lead to a significant decrease of P4 to basal levels after 24 h. The latter might mirror the slow increase in placental PGF2α in cats, with metabolite PGFM becoming measurable at three weeks prior to parturition. Prostaglandins are more effective during the second half of gestation.

When two injections of the antiprogesterone aglepristone were given from day 21 to 22, or from day 35 to 38 and on, abortion occurred in all cats [43]). Two injections of aglepristone given later, on days 45 and 46, caused abortion in four-sixths of cats. Progesterone values remained high during abortion but decreased towards day 55 [45]. And in another study, two injections of aglepristone given on day 33.3 on average caused abortion in 54/61 of cats [46]. These studies highlight the impact of receptor-bound progesterone on maintenance of pregnancy.

Prolactin affects luteotropic from mid-gestation on, and the dopamine agonist cabergoline antagonizes the effect. Daily injections of cabergoline for 5 days from day 30 on caused abortion in four-fifths of cats after a decrease of progesterone to <1 ng/mL [34]. Daily oral applications between days 34 and 42 until abortion caused abortion in all cats [47]. Similarly, daily oral cabergoline application between days 36 and 40.8 induced abortion in all animals; however, the same treatment starting at day 48.5 of gestation induced premature parturition [48]. In another study, daily oral application starting at days 45 and 47 similarly failed to induce abortion in two cats [47]. Daily application of cabergoline from day 30 on for 11 days and the application of PGF2α every other day led to 100% of abortions. The same protocol used between days 25 and 47 showed the same effect [49]. Dopamine agonists are apparently highly effective from mid-gestation on and even without PG and are less effective during late gestation. However, combined with PG, earlier induction of abortion may be successful. More details concerning the application schemes are given in Table 1.

## 6. Physiological Mechanisms in the Oviduct and Uterus—What Is Different from Dogs

In cats, the early zygote enters the uterine lumen by days 5–6 after coitus, a few days earlier than in dogs, but similarly in the morula stage. The crosstalk between the oviduct and dividing embryo during the passage through the oviduct is currently under investigation. The feline ovarian epithelium secretes extracellular vesicles (EVs) that influence sperm function and fertilization and are incorporated into the embryo, probably affecting embryo development, as in humans [16,50,51]. These small exosomes and microvesicles are approximately 40–150 nm in diameter and contain an abundance of DNA, RNA, proteins and probably more; they are released from the inner cell (exosomes) or the cell membrane (microvesicles) and support cell–cell communication via the release of their contents [50]. It is not known whether the feline embryo or endometrium secrete EV as in other species [52].

After arrival in the uterus around day 6–8 after fertilization, cat embryos develop and migrate within and between uterine horns (spacing) for even distribution. At day 7, most embryos have reached the blastocyst stage. Around day 10, the surface epithelium appears folded, and the endometrial glands show beginning secretion. Until day 12, endometrial cellular and tissue changes occur under the influence of estrogen and progesterone, which are comparable with the canine species and prepare the endometrium for the arrival of the embryos. Estrogens induce proliferation of endometrial and stromal cells, angiogenesis and upregulation of E2 and P4 receptors. Formation of CLs leads to increasing P4 concentrations, inhibiting proliferation, and the endometrium enters the secretory phase. The embryo then undergoes the phases of apposition and adhesion; blastocysts loose the zona pellucida and expand, and some start to secrete [40,53]. Cytoplasmic protrusions emerge from the early embryo to contact the local swellings and edematous sites of the endometrium, the implantation sites. Thereafter, around day 14, penetration of the endometrium by trophoblast cell columns begins, and implantation is completed by day 20, with consecutive formation and differentiation of the zonary/girdle placenta, which is endotheliochorial in cats; these events occur a few days earlier than in dogs [54]. In one study, an incomplete zonary placenta was found in 62.5% of pregnant cats, distal to the insertion of the umbilical cord [55]; in dogs, the zonary placenta completely surrounds the fetus. In cats, decidualization of endometrial and stromal cells is currently under investigation. Decidualization describes the morphological and functional change in these cells, undergoing proliferation and differentiation into secretory cells and expressing specific markers like growth factors and hormone receptors. In vitro studies using feline endometrial and stromal cells showed that under the influence of P4 and E2, morphological decidualization of cells occurred; however, some decidualization markers like vascular endothelial growth factor (VEGF) or progesterone receptor were not upregulated [56] as during comparable studies in dogs [57]. The search for species-specific decidualization markers is ongoing.

## 7. Physiological Biomolecular Mechanisms at the Uterine Level

Platelet-activating factor (PAF), an angiogenetic and proliferative agent, is an important mediator during fertilization, implantation and parturition in many species (mice, humans). In dogs, PAF and receptor genes were found to be significantly elevated in the canine uterus/placenta during the pre-implantation period [58], which is under the control of progesterone. This was not confirmed in the feline uterus/placenta [59]. But like in dogs and other species, markers of endometrial receptivity were detected; transcripts for growth factors, cytokines and enzymes, contributing to degradation of the extracellular matrix, angiogenesis, growth, immunomodulation and decidualization of stromal cells (GM-CSF, IFNγ, MMP2, IGFI-II and receptors, EGF, TGF-β, IGF-IIR), were found to be upregulated in the feline uterus/placenta in the peri-implantation period [38,60]. Increasing expression of IGF-II, EGF and MMP-2 towards the post-implantation period was found, and expression of IFNγ significantly increased towards mid-gestation. TGF-β and GM-CSF were constantly expressed until mid-gestation. The function and complex interplay are not sufficiently investigated in cats. Hypoxia-inducible factor (HIF) and vascular endothelial growth factor (VEGF) genes were assessed in the feline uterus/placenta [37,61]. A hypoxic environment is a prerequisite for the development of pregnancy. HIFs regulate placentation and vascularization by the regulation of VEGF expression. Expression at placentation sites increased towards late gestation, which was paralleled by expression of VEGF [37]. In a recent study, placental protein and gene expression of VEGF-A and other members of the VEGF-A family were examined throughout feline pregnancy; protein and gene expression was found to be differentially upregulated at different time points during gestation within the maternal and fetal microvasculature and endothelial and trophoblast cells. The study highlights the importance of VEGF for vascularization and normal placenta development in cats [61].

## 8. Sonographical Monitoring of the Developing Pregnancy

Earliest recognition of gestational sacs as anechoic regions with hyperechoic borders of 2 mm in diameter was on day 10 in one study [62]. During the embryo phase, the gestational sacs are round until approximately day 20, becoming slightly ovoid thereafter [63]. The diameter continuously increases until it reaches approximately 35.7–37 mm in diameter between days 29 and 30. The embryo becomes detectable between days 15 and 17 as a hyperechoic spot attached to the gestational chamber wall; the heartbeat becomes visible at approximately day 16. At day 17, the embryo appears separated from the wall. Fetal membranes become differentiated around day 20, surrounding the embryo, which is located in the center of the gestational sac [64,65]. The length of the embryo was described to be 3.3 mm at day 17, 8.7 mm at day 20, 11.2 mm at day 22, 17.4 mm at day 25 and 30.1 mm at day 30 [62].

**Figure 1:**
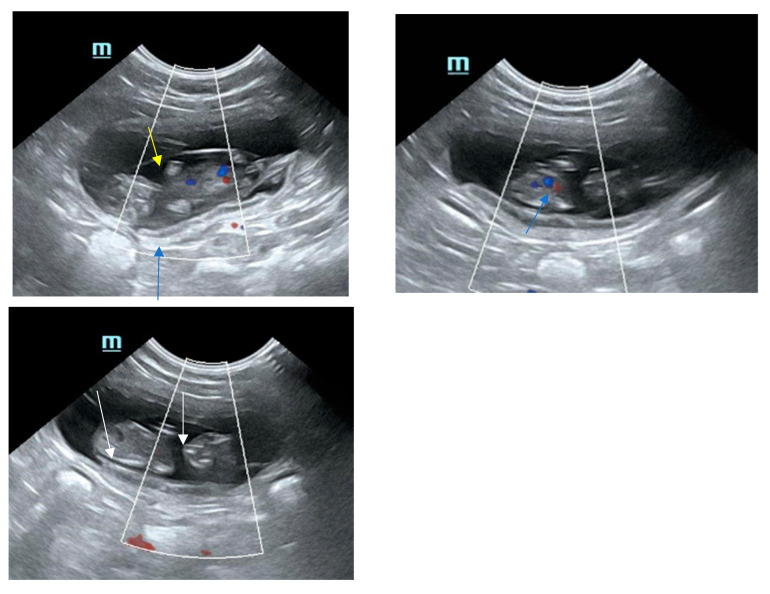
Sonographical pictures of a feline gestational sac with fetus at day 37. Note the fine, hyperechoic, inner line of the placenta (blue arrow), the hypoechoic fetal membranes (yellow arrow) and the decent calcification of the fetus (white arrows). Feline fetus, day 37 of gestation.

During the second half of pregnancy, the biparietal diameters becomes >15 mm. Detailed information on ultrasonographic biometry during fetal development are given in previous studies [5,62,63,66,67,68]. The uterine layers can be well differentiated; the serosa is a thin, hyperechoic outer rim, the echogenicity of the myometrium appears intermediate, hypoechoic to echoic, and the inner layer, the endometrium, appears as hypoechoic layer with multiple hyperechoic spots. The sonographical appearance of the placenta is dependent on developmental stage. Whereas in the embryo phase, the layers are more homogenous and difficult to differentiate, in the fetal phase, the thickening layer looks structured with increasing vascularization, with an inner and outer hyperechoic thin line and an inner hypoechoic line. The fetal membranes appear hypoechoic and become well visible after day 30 (yolk sac, amnion and allantois; see Figure 1). The thickness of the uterine wall ranged from 2.4 to 6.8 mm in one study [69]. Inner organs can be differentiated from day 30 on; the layered structure of the intestine is visible late, between days 52 and 56 [65,70].

## 9. Outlook

More studies are needed on embryo–maternal crosstalk, extracellular vesicles, feline decidualization, the function of AMH and CRH and the initiation of parturition in cats. In vivo studies throughout gestation are needed.

## 10. Conclusions

This review provides an overview over recent achievements on the establishment of pregnancy in cats. While many details are available on ovarian function and placental secretory activity, information on the complex changings of the endometrium and uterus in the course of pregnancy is scarce, even though very recent studies tried to establish endometrial organoids from endometrial epithelial stem cells [71]. Some important gene activities could be detected even after freezing thawing; however, these models are lacking the in vivo milieu and only mirror specific time points. Furthermore, the culture conditions are under investigation; therefore, these models at present can only give preliminary and uncomplete insights into endometrial function.

## Figures and Tables

**Table 1:** Effect of suppression of steroidogenic hormones on the course of feline gestation.

Measure	Dosages	Day of Gestation	Nr of Cats That Aborted/Total Nr of Treated Cats	Authors
OE		35	5/5	Tsutsui et al., 2009 [41]
OE		40	4/5	Tsutsui et al., 2009 [41]
OE		45	2/5	Tsutsui et al., 2009 [41]
OE		50	3/5	Tsutsui et al., 2009 [41]
Cloprostenol	5 µg/kg s.c. on three days	21–22	0/6	Garcia-Mitacek et al., 2012 [42]
Cloprostenol	5 µg/kg s.c. on three days	21–22	0/10	Garcia-Mitacek et al., 2017 [43]
Cloprostenol	5 µg/kg s.c. on three days	35–38	1/7	Garcia-Mitacek et al., 2012 [42]
Cloprostenol	5 µg/kg s.c. on three days	35–38	1/10	Garcia-Mitacek et al., 2017 [43]
Natural PGF2α	2 mg per cat s.c.	33	4/4	Verstegen et al., 1993 [34]
Aglepristone	10 mg/kg s.c. on two days	21 + 25 or 35 + 36	10/10 10/10	Garcia-Mitacek et al., 2017 [43]
Aglepristone	10 mg/kg s.c. on two days	45	4/6	Georgiev et al., 2010 [45]
Aglepristone	15 mg/kg s.c. on two days	33.3 ± 4.2 days	88.5% (54/61)	Fieni et al., 2006 [46]
Caberboline Injections	1.65 µg/kg/day s.c. for 5 d	Daily from day 30 on	80% (4/5)	Verstegen et al., 1993 [34]
Caberboline	15 µg/kg orally	Daily between d 34 and 42 (until abortion) Daily starting between d 45 and 47	100% (8/8) Failure in two cats	Erünal-Maral et al., 2004 [47]
Caberboline Oral appl.	5–15 µg/kg orally	Daily between d 36 ± 6.17 and 40.8 ± 6.96 Daily starting at on average d 48.5	100% (41/41) Premature parturition	Jöchle and Jöchle 1988 [48]
Cabergoline + prostagl.	5 µg/kg orally 5 µg/kg s.c. every other day	Daily from day 30 on (11 d until abortion), PG every other day	100%	Onclin and Verstegen 1997 [49]
Cabergoline + prostagl.	5 µg/kg orally 5 µg/kg s.c. every other day	Daily from day 25–47 on (11 d until abortion), PG every other day	100%	Onclin and Verstegen 1997 [49]

## Data Availability

Not applicable.
